# Avascular Necrosis of the Femoral Head Following Intramedullary Nailing of a Femoral Shaft Fracture in a Skeletally Mature Adult: A Case Report

**DOI:** 10.7759/cureus.53357

**Published:** 2024-01-31

**Authors:** Hamza R Gomaa, Ali A Buanq, Ahmed M Hazeem, Salma M Daoud, Ali H Almajed

**Affiliations:** 1 Orthopedics and Neurosurgery, Bahrain Defense Force Royal Medical Services, Riffa, BHR; 2 General Practice, Albaraka Fertility Hospital, Manama, BHR

**Keywords:** femoral nailing, skeletally mature, intramedullary femur nailing, femoral shaft fractures, hip avascular necrosis

## Abstract

Avascular necrosis (AVN) of the femoral head is a well-documented complication that occurs following femoral neck fractures in both adults and pediatrics. Incidence of AVN following intramedullary nailing (IMN) for femoral shaft fractures is relatively rare. We are reporting an exceptional case of a 28-year-old skeletally mature adult, with no risk factors, who developed stage 3 AVN following trochanteric entry-point IMN for a traumatic femur shaft fracture. Our case contributes to the existing literature by adding to the limited number of reported cases available. In addition, it emphasizes the importance of observation and anticipation for such complications and shows the need for further studies to understand the relationship between this modality of treatment and the development of AVN of the femoral head.

## Introduction

Femoral shaft fractures are frequently encountered following high-velocity traumas, demanding effective management strategies to optimize functional outcomes and mitigate complications [1]. Intramedullary nailing (IMN) has emerged as the gold standard for the treatment of isolated femoral shaft fractures, boasting high union rates and a low risk of infection [2]. Yet, IMN is not immune to complications, with potential adverse events encompassing neurovascular injury, compartment syndrome, malalignment, and delayed union [3]

Avascular necrosis (AVN) of the femoral head, conventionally associated with intracapsular femoral neck fractures [4], has been identified as an unusual complication following IMN in pediatric populations [5]. In contrast to previous cases mostly involving the piriformis fossa as the entry point [6,7,8], our case challenges the usual patterns and highlights the complexities in understanding and treating AVN in adults who underwent IMN for a femur fracture. The patient's decision not to undergo surgery made the treatment plan more complex. This case adds valuable information to our understanding of complications from femur fractures, emphasizing the need for careful thinking and ongoing research to fully understand and manage such cases.

## Case presentation

**Figure 1:**
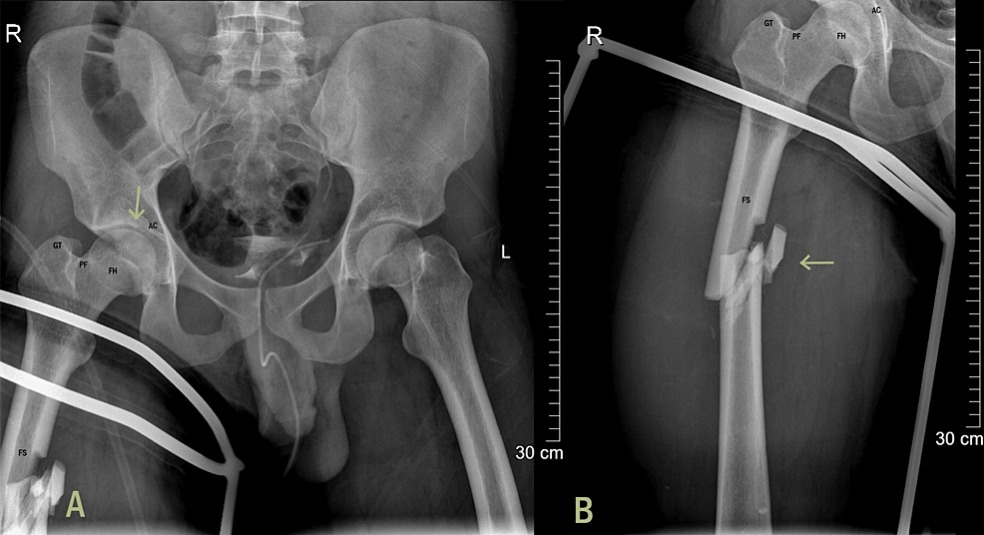
(A) Pre-op AP pelvis and femur X-rays demonstrating the absence of AVN at the femoral head at the time of initial injury; (B) isolated femoral shaft fracture with no involvement of intracapsular proximal femur. FH, femoral head; AC, acetabulum; PF, piriformis fossa; GT, greater trochanter; FS, femoral shaft; AVN, avascular necrosis; AP, anteroposterior

Our patient was a 28-year-old male who had no known medical illnesses and no history of corticosteroid use, alcohol consumption, smoking, or radiation therapy. He sustained a multi-vehicle collision at 23 years of age, resulting in an isolated closed shaft femur fracture with no involvement of the neck of femur or any associated intra-articular hip injuries. There was no evidence of preexisting AVN of the femoral head on the anteroposterior pelvis X-ray taken in the emergency department following the injury (Figures 1A-1B).

**Figure 2:**
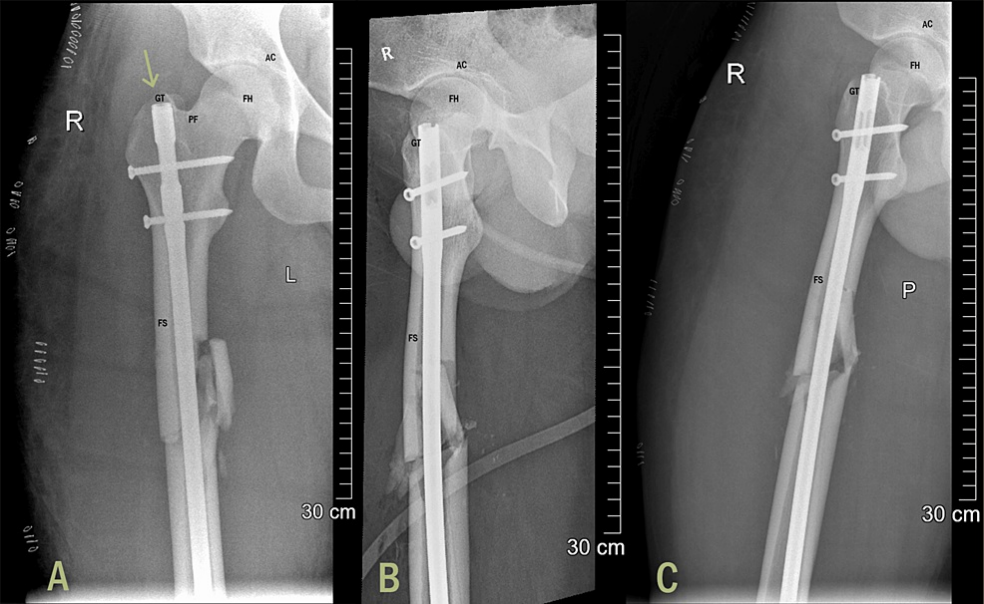
Immediate post-op images at the time of fixation showing the nail inserted using the trochanteric entry point: (A) AP view, (B) lateral view, and (C) oblique view. FH, femoral head; AC, acetabulum; PF, piriformis fossa; GT, greater trochanter; FS, femoral shaft

After stabilization, the patient underwent closed reduction and internal fixation for a femoral shaft fracture using an antegrade intramedullary nail inserted through the greater trochanter entry point (Figures 2A-2C).

Following the surgery, the patient was mobilized with physiotherapy and discharged home in stable condition. Outpatient follow-up visits following the operation were uneventful. He presented five years later, at the age of 28, with newly developed right hip pain persisting for around three months and associated with a painful hip range of motion. The patient stated that the pain was exacerbated by activity and relatively relieved using analgesics. There was no history of recurrent hip trauma following the initial injury.

Upon physical examination, the patient was mobilizing full weight-bearing unassisted but with an antalgic gait. On inspection, there were no signs of local inflammation or swelling. The patient had no local bone tenderness over the hip region. He showed restricted internal and external rotation compared to the opposite side due to pain. However, the hip's full range of motion in flexion, adduction, and abduction was achieved. The distal neurovascular function was intact, with good pulses and sensation. Motor power was 5/5.

**Figure 3:**
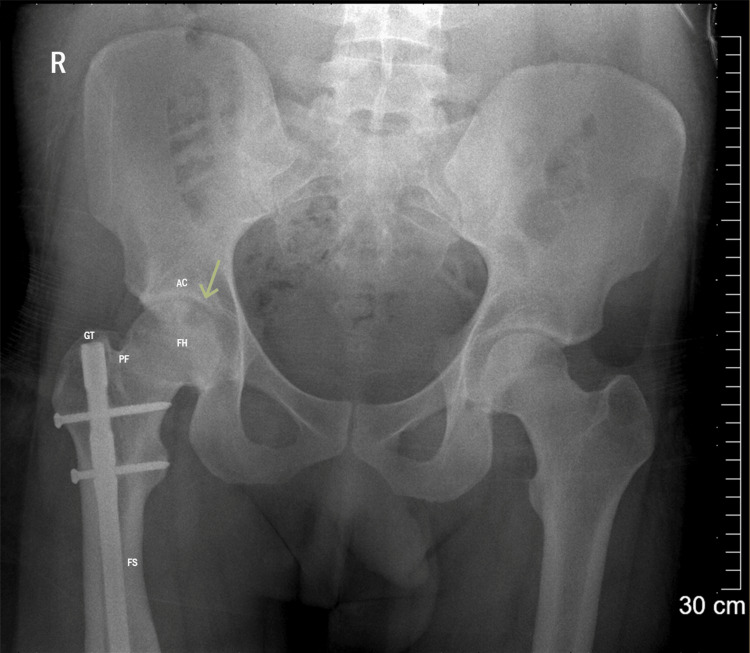
Pelvic X-rays obtained five years post-surgery showing AVN of the femoral head with a crescent sign and cortical collapse. FH, femoral head; AC, acetabulum; PF, piriformis fossa; GT, greater trochanter; FS, femoral shaft; AVN, avascular necrosis

A plain hip X-ray conducted at the presentation showed evidence of AVN of the femoral head (Ficat grade 3), as shown in Figure 3.

**Figure 4:**
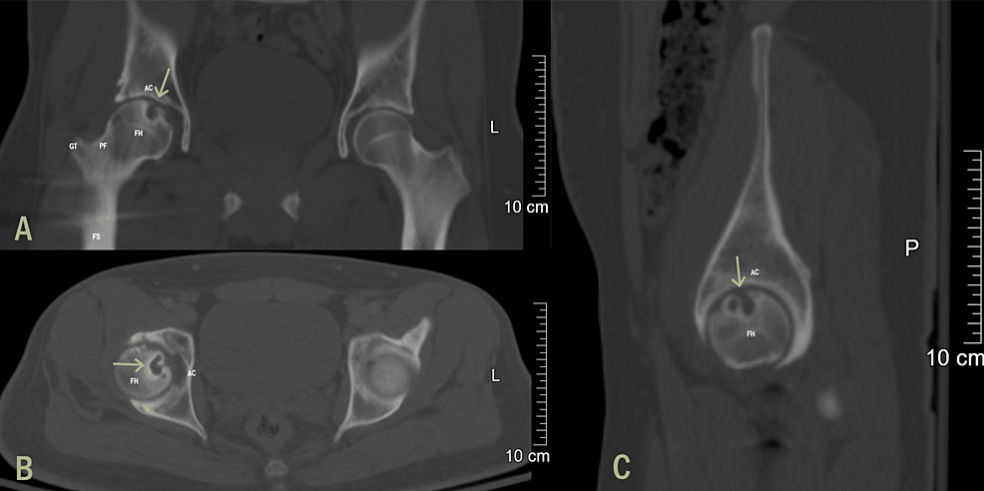
CT scan confirming AVN of the femoral head: (A) coronal plane, (B) axial plane, and (C) sagittal plane. FH, femoral head; AC, acetabulum; PF, piriformis fossa; GT, greater trochanter; FS, femoral shaft; AVN, avascular necrosis

A CT scan was conducted and confirmed the diagnosis of AVN grade 3 with early femoral head cortical collapse (Figure 4).

The patient was offered surgical treatment options, including core decompression or total hip replacement; however, he was not willing to undergo surgical intervention at that time.

## Discussion

The case we presented involved a 28-year-old skeletally mature adult with no history of preexisting AVN of the femoral head and no preexisting risk factors. The patient presented long after the initial femoral shaft fracture with long-standing hip pain and a limitation of range of motion, which was not attributed to the initial trauma or any subsequent traumas. Investigations, including X-ray and CT scan, showed stage 3 AVN of the femoral head with subchondral collapse and crescent sign. The only risk factor for developing such a lesion in our patient based on history was the intramedullary nailing done following a traumatic femur shaft fracture. Intramedullary nailing was done through a trochanteric entry point rather than the piriformis fossa. This adds to the uniqueness of the case. 

This case contributes to the existing literature by expanding the understanding of AVN following intramedullary nailing, specifically in skeletally mature adults with an entry point at the greater trochanter. Such contributions are essential for improving clinical awareness, refining surgical techniques, and enhancing patient outcomes.

Femur fracture is a common injury following high-velocity traumas [1]. Intramedullary nailing is the standard of care for isolated femoral shaft fractures [1]. Antegrade intramedullary femur nailing was associated with up to 98% to 99% union rates and a low risk of infection (1%-2%) even when used in open fractures [2]. Apart from infection, other potential complications include neurovascular injury, compartment syndrome, malalignment, nonunion, malunion, and delayed union [3].

AVN of the femoral head, although a common complication following intracapsular femoral neck fractures [4], is a documented complication following intramedullary nailing of femur fractures in children and adolescents [5]. Multiple cases of AVN of the femoral head following intramedullary nailing of femoral shaft fractures were reported in English literature. However, all cases reported involved patients younger than 14 years who were skeletally immature [5-7,9-10] except for one case reported in 2008 by Graves et al. [11]. 

To understand the etiology of AVN, it is crucial to understand the anatomy of the hip [12]. The blood supply to the femoral head comes mainly from the medial femoral circumflex artery (MFCA) and its branches, namely the posterosuperior and posteroinferior reticular artery [13]. After skeletal maturity, the main branch supplying the femoral head is the lateral epiphyseal branch of the MFCA [13]. AVN of the femoral head following IMN for the femoral shaft is believed to be caused by traumatic injury to these arteries, mainly the posterosuperior branch, which is near the insertion point of the nail at the piriformis [12]. The severity of the traumatic injury also depends on several factors such as age, sex, types of trauma, and developmental patterns [14]. 

In 2016, Kim et al. studied the incidence of AVN of the femoral head after intramedullary nailing of femoral shaft fractures through a multicenter retrospective analysis of 542 cases [5]. The study included all patients with femoral shaft fractures treated with antegrade interlocking IMN [5]. They excluded patients with risk factors for AVN, including immunosuppressive medications intake, pathological fractures, femoral neck fractures, femoral shaft fractures reaching the inter- or subtrochanteric region, divers, and patients with less than one year of follow-up [5]. It was concluded that the overall incidence of AVN was 0.2%, with a 4% incidence in patients with an open physis, compared to 0% in patients with a closed physis. The incidence of AVN was 1.1% in patients less than 20 years of age compared to 0% in those over 20. There were no reported cases of AVN following intramedullary nailing of isolated femoral shaft fractures in patients aged >20 years regardless of the entry point [5].

All reported cases of AVN following IMN in adolescents were reported using piriformis entry points [5,6,7,9]. Only one case was reported following the trochanteric entry point by Stans et al. [15]. All these cases were reported in pediatric and adolescent patients. The only case of AVN reported following IMN of a femur fracture in a skeletally mature adult aged 16 years was by Graves and Sands [11]. However, in this case, the nail was inserted using the piriformis fossa. Additionally, it was reported following the removal of the IMN rather than the insertion itself, making it difficult to determine the exact contributing factor for the development of AVN in their case.

Our patient was having continuous hip pain affecting his mobility and range of motion. These symptoms started a couple of years following the intramedullary nail fixation, which could be attributed to the AVN. Other possible causes of hip pain include soft tissue sprains, contusions, iliopsoas bursitis, osteoarthritis, developmental anomalies, accessory ossicles, anatomical variations, or fractures [14,16,17]. In addition, he did not undergo the removal of the implant or any other interventions. The patient was offered surgical hip replacement versus core decompression surgery, but he refused surgical intervention and opted for physiotherapy and pain management.

## Conclusions

In conclusion, the presented case underlines the atypical occurrence of AVN of the femoral head in a skeletally mature adult following IMN through a trochanteric entry point for traumatic femur shaft fracture. Notably, this case deviates from the predominant instances reported in the literature, where AVN post-IMN primarily affects adolescents, typically utilizing piriformis entry points.

This unique case highlights the necessity for continued vigilance regarding potential AVN as a complication arising from the initial IMN fixation procedure. Furthermore, the inclusion of this case in the existing literature serves to broaden the spectrum of reported occurrences, providing a valuable contribution by showing the manifestation of AVN in skeletally mature adults and emphasizing the importance of anticipation of such complications in the management of femoral shaft fractures. Further research and accumulated clinical experiences will contribute to a better understanding of the risk factors and optimal management strategies for AVN in skeletally mature individuals following IMN for femoral shaft fractures.
